# Supplementary material for: Movies reveal the fine-grained organization of infant visual cortex
Source: eLife. 2025 Mar 6;12:RP92119. doi: 10.7554/eLife.92119 (PMC11884787; doi:10.7554/eLife.92119)
Supplement: Peer review history

# Editor report: eLife Assessment

**DOI:** 10.7554/eLife.92119.4.sa0

**Authors:** Jessica Dubois

**Affiliations:** Inserm Unité NeuroDiderot, Université Paris Cité France

**Keywords:** Compelling, Convincing, Valuable

This study presents **valuable** evidence concerning the potential for naturalistic movie-viewing fMRI experiments to reveal some features that are correlated with the functional and topographical organization of the developing visual system in awake infants and toddlers. The data are **compelling** given the difficulty of studying this population, the methodology is original and validated, and the evidence supporting the conclusions is **convincing** and in line with prior research using resting-state and awake task-based fMRI. This study will be of interest to cognitive neuroscientists and developmental psychologists, and in particular those interested in using fMRI to investigate brain organisation in pediatric and clinical populations with limited tolerance to fMRI.

# Referee report: Reviewer #2 (Public review)

**DOI:** 10.7554/eLife.92119.4.sa1

Summary:

This manuscript reports analyses of fMRI data from infants and toddlers watching naturalistic movies. Visual areas in the infant brain show distinct functions, consistent with previous studies using resting state and awake task-based infant fMRI. The pattern of activity in visual regions contains some features predicted by the regions' retinotopic responses. The revised version of the manuscript provides additional validation of the methodology, and clarifies the claims. As a result, the data provide clear support for the claims.

Strengths:

The authors have collected a unique dataset: the same individual infants both watched naturalistic animations and a specific retinotopy task. Using these data position the authors show that activity evoked by movies, in infants' visual areas, is correlated with the regions' retinopic response. The revised manuscript validates this methodology, using adult data. The revised manuscript also shows that an infant's movie watching data is not sufficient or optimal to predict their visual areas' retinotopic responses; anatomical alignment with a group of previous participants provides more accurate prediction of a new participant's retinotopic response.

Weaknesses:

A key step in the analysis of the movie-watching data is the selection of independent components of the movie evoked response that resemble retinotopic spatial patterns. While the trained researcher was unlikely to be biased by this infant's own retinotopy, he/she was actively looking for ICs that resemble average patterns of retinotopic response. To show that these ICs didn't arise by chance (i.e. in noise), the authors proposed an additional analysis in the revised manuscript, by misaligning the functional and anatomical data for a subset of participants. This only partially confirms the reliability of the original components, since when the (new) coder tried to be conservative to avoid false components, he/she identified just over half of the 'true' components (13 vs 22 estimated over the group of 6 infants).

# Author comment: Author response

**DOI:** 10.7554/eLife.92119.4.sa2

**Authors:** Cameron T Ellis, Tristan S Yates, Michael J Arcaro, Nicholas Turk-Browne

**Affiliations:** Stanford University Palo Alto United States; Columbia University New York United States; University of Pennsylvania Philadelphia United States; Yale University New Haven United States

The following is the authors’ response to the previous reviews.

**eLife Assessment**

This study presents valuable findings on the potential of short-movie viewing fMRI protocol to explore the functional and topographical organization of the visual system in awake infants and toddlers. Although the data are compelling given the difficulty of studying this population, the evidence presented is incomplete and would be strengthened by additional analyses to support the authors' claims. This study will be of interest to cognitive neuroscientists and developmental psychologists, especially those interested in using fMRI to investigate brain organisation in pediatric and clinical populations with limited fMRI tolerance.

We are grateful for the thorough and thoughtful reviews. We have provided point-bypoint responses to the reviewers’ comments, but first, we summarize the major revisions here. We believe these revisions have substantially improved the clarity of the writing and impact of the results.

Regarding the framing of the paper, we have made the following major changes in response to the reviews:

(1) We have clarified that our goal in this paper was to show that movie data contains topographic, fine-grained details of the infant visual cortex. In the revision, we now state clearly that our results should not be taken as evidence that movies could replace retinotopy and have reworded parts of the manuscript that could mislead the reader in this regard.

(2) We have added extensive details to the (admittedly) complex methods to make them more approachable. An example of this change is that we have reorganized the figure explaining the Shared Response Modelling methods to divide the analytic steps more clearly.

(3) We have clarified the intermediate products contributing to the results by adding 6 supplementary figures that show the gradients for each IC or SRM movie and each infant participant.

In response to the reviews, we have conducted several major analyses to support our findings further:

(1) To verify that our analyses can identify fine-grained organization, we have manually traced and labeled adult data, and then performed the same analyses on them. The results from this additional dataset validate that these analyses can recover fine-grained organization of the visual cortex from movie data.

(2) To further explore how visual maps derived from movies compare to alternative methods, we performed an anatomical alignment control analysis. We show that high-quality maps can be predicted from other participants using anatomical alignment.

(3) To test the contribution of motion to the homotopy analyses, we regressed out the motion effects in these analyses. We found qualitatively similar results to our main analyses, suggesting motion did not play a substantial role.

(4) To test the contribution of data quantity to the homotopy analyses, we correlated the amount of movie data collected from each participant with the homotopy results. We did not find a relationship between data quantity and the homotopy results.

**Public Reviews:**

**Reviewer #1 (Public Review):**

Summary:

Ellis et al. investigated the functional and topographical organization of the visual cortex in infants and toddlers, as evidenced by movie-viewing data. They build directly on prior research that revealed topographic maps in infants who completed a retinotopy task, claiming that even a limited amount of rich, naturalistic movie-viewing data is sufficient to reveal this organization, within and across participants. Generating this evidence required methodological innovations to acquire high-quality fMRI data from awake infants (which have been described by this group, and elsewhere) and analytical creativity. The authors provide evidence for structured functional responses in infant visual cortex at multiple levels of analyses; homotopic brain regions (defined based on a retinotopy task) responded more similarly to one another than to other brain regions in visual cortex during movie-viewing; ICA applied to movie-viewing data revealed components that were identifiable as spatial frequency, and to a lesser degree, meridian maps, and shared response modeling analyses suggested that visual cortex responses were similar across infants/toddlers, as well as across infants/toddlers and adults. These results are suggestive of fairly mature functional response profiles in the visual cortex in infants/toddlers and highlight the potential of movie-viewing data for studying finer-grained aspects of functional brain responses, but further evidence is necessary to support their claims and the study motivation needs refining, in light of prior research.

Strengths:

- This study links the authors' prior evidence for retinotopic organization of visual cortex in human infants (Ellis et al., 2021) and research by others using movie-viewing fMRI experiments with adults to reveal retinotopic organization (Knapen, 2021).

- Awake infant fMRI data are rare, time-consuming, and expensive to collect; they are therefore of high value to the community. The raw and preprocessed fMRI and anatomical data analyzed will be made publicly available.

We are grateful to the reviewer for their clear and thoughtful description of the strengths of the paper, as well as their helpful outlining of areas we could improve.

Weaknesses:

- The Methods are at times difficult to understand and in some cases seem inappropriate for the conclusions drawn. For example, I believe that the movie-defined ICA components were validated using independent data from the retinotopy task, but this was a point of confusion among reviewers.

We acknowledge the complexity of the methods and wish to clarify them as best as possible for the reviewers and the readers. We have extensively revised the methods and results sections to help avoid potential misunderstandings. For instance, we have revamped the figure and caption describing the SRM pipeline (Figure 5).

To answer the stated confusion directly, the ICA components were derived from the movie data and validated on the (completely independent) retinotopy data. There were no additional tasks. The following text in the paper explains this point:

“To assess the selected component maps, we correlated the gradients (described above) of the task-evoked and component maps. This test uses independent data: the components were defined based on movie data and validated against task-evoked retinotopic maps.” Pg. 11

In either case: more analyses should be done to support the conclusion that the components identified from the movie reproduce retinotopic maps for example, by comparing the performance of movie-viewing maps to available alternatives (anatomical ROIs, group-defined ROIs).

Before addressing this suggestion, we want to restate our conclusions: features of the retinotopic organization of infant visual cortex could be predicted from movie data. We did not conclude that movie data could ‘reproduce’ retinotopic maps in the sense that they would be a replacement. We recognize that this was not clear in our original manuscript and have clarified this point throughout, including in this section of the discussion:

“To be clear, we are not suggesting that movies work well enough to replace a retinotopy task when accurate maps are needed. For instance, even though ICA found components that were highly correlated with the spatial frequency map, we also selected some components that turned out to have lower correlations. Without knowing the ground truth from a retinotopy task, there would be no way to weed these out. Additionally, anatomical alignment (i.e., averaging the maps from other participants and anatomically aligning them to a held-out participant) resulted in maps that were highly similar to the ground truth. Indeed, we previously(Henriksson et al., 2008) found that adult-defined visual areas were moderately similar to infants. While functional alignment with adults can outperform anatomical alignment methods in similar analyses (Lu et al., 2017), here we find that functional alignment is inferior to anatomical alignment. Thus, if the goal is to define visual areas in an infant that lacks task-based retinotopy, anatomical alignment of other participants’ retinotopic maps is superior to using movie-based analyses, at least as we tested it.” Pg. 21

As per the reviewer’s suggestion and alluded to in the paragraph above, we have created anatomically aligned visual maps, providing an analogous test to the betweenparticipant analyses like SRM. We find that these maps are highly similar to the ground truth. We describe this result in a new section of the results:

“We performed an anatomical alignment analog of the functional alignment (SRM) approach. This analysis serves as a benchmark for predicting visual maps using taskbased data, rather than movie data, from other participants. For each infant participant, we aggregated all other infant or adult participants as a reference. The retinotopic maps from these reference participants were anatomically aligned to the standard surface template, and then averaged. These averages served as predictions of the maps in the test participant, akin to SRM, and were analyzed equivalently (i.e., correlating the gradients in the predicted map with the gradients in the task-based map). These correlations (Table S4) are significantly higher than for functional alignment (using infants to predict spatial frequency, anatomical alignment > functional alignment: ∆_Fisher Z_ M=0.44, CI=[0.32–0.58], p<.001; using infants to predict meridians, anatomical alignment > functional alignment: ∆_Fisher Z_ M=0.61, CI=[0.47–0.74], p<.001; using adults to predict spatial frequency, anatomical alignment > functional alignment: ∆_Fisher Z_ M=0.31, CI=[0.21–0.42], p<.001; using adults to predict meridians, anatomical alignment > functional alignment: ∆_Fisher Z_ M=0.49, CI=[0.39–0.60], p<.001). This suggests that even if SRM shows that movies can be used to produce retinotopic maps that are significantly similar to a participant, these maps are not as good as those that can be produced by anatomical alignment of the maps from other participants without any movie data.” Pg. 16–17

Also, the ROIs used for the homotopy analyses were defined based on the retinotopic task rather than based on movie-viewing data alone - leaving it unclear whether movie-viewing data alone can be used to recover functionally distinct regions within the visual cortex.

We agree with the reviewer that our approach does not test whether movie-viewing data alone can be used to recover functionally distinct regions. The goal of the homotopy analyses was to identify whether there was functional differentiation of visual areas in the infant brain while they watch movies. This was a novel question that provides positive evidence that these regions are functionally distinct. In subsequent analyses, we show that when these areas are defined anatomically, rather than functionally, they also show differentiated function (e.g., Figure 2). Nonetheless, our intention was not to use the homotopy analyses to define the regions. We have added text to clarify the goal and novelty of this analysis.

“Although these analyses cannot define visual maps, they test whether visual areas have different functional signatures.” Pg. 6

Additionally, even if the goal were to define areas based on homotopy, we believe the power of that analysis would be questionable. We would need to use a large amount of the movie data to define the areas, leaving a low-powered dataset to test whether their function is differentiated by these movie-based areas.

- The authors previously reported on retinotopic organization of the visual cortex in human infants (Ellis et al., 2021) and suggest that the feasibility of using movie-viewing experiments to recover these topographic maps is still in question. They point out that movies may not fully sample the stimulus parameters necessary for revealing topographic maps/areas in the visual cortex, or the time-resolution constraints of fMRI might limit the use of movie stimuli, or the rich, uncontrolled nature of movies might make them inferior to stimuli that are designed for retinotopic mapping, or might lead to variable attention between participants that makes measuring the structure of visual responses across individuals challenging. This motivation doesn't sufficiently highlight the importance or value of testing this question in infants. Further, it's unclear if/how this motivation takes into account prior research using movie-viewing fMRI experiments to reveal retinotopic organization in adults (e.g., Knapen, 2021). Given the evidence for retinotopic organization in infants and evidence for the use of movie-viewing experiments in adults, an alternative framing of the novel contribution of this study is that it tests whether retinotopic organization is measurable using a limited amount of movie-viewing data (i.e., a methodological stress test). The study motivation and discussion could be strengthened by more attention to relevant work with adults and/or more explanation of the importance of testing this question in infants (is the reason to test this question in infants purely methodological - i.e., as a way to negate the need for retinotopic tasks in subsequent research, given the time constraints of scanning human infants?).

We are grateful to the reviewer for giving us the opportunity to clarify the innovations of this research. We believe that this research contributes to our understanding of how infants process dynamic stimuli, demonstrates the viability and utility of movie experiments in infants, and highlights the potential for new movie-based analyses (e.g., SRM). We have now consolidated these motivations in the introduction to more clearly motivate this work:

“The primary goal of the current study is to investigate whether movie-watching data recapitulates the organization of visual cortex. Movies drive strong and naturalistic responses in sensory regions while minimizing task demands (Nastase et al., 2020; Finn et al., 2022; Ellis et al., 2021) and thus are a proxy for typical experience. In adults, movies and resting-state data have been used to characterize the visual cortex in a data-driven fashion (Loiotile et al., 2019; Knapen, 2021; Lu et al., 2017). Movies have been useful in awake infant fMRI for studying event segmentation (Guntupalli et al., 2016), functional alignment (Yates et al., 2022), and brain networks (Turek et al., 2018). However, this past work did not address the granularity and specificity of cortical organization that movies evoke. For example, movies evoke similar activity in infants in anatomically aligned visual areas (Guntupalli et al., 2016), but it remains unclear whether responses to movie content differ between visual areas (e.g., is there more similarity of function within visual areas than between [Yates et al., 2023]). Moreover, it is unknown whether structure within visual areas, namely visual maps, contributes substantially to visual evoked activity. Additionally, we wish to test whether methods for functional alignment can be used with infants. Functional alignment finds a mapping between participants using functional activity – rather than anatomy – and in adults can improve signal-to-noise, enhance across participant prediction, and enable unique analyses (Lu et al., 2017; Li et al., 2022; Busch et al., 2021; Chen et al., 2025)].” Pg. 3-4

Furthermore, the introduction culminates in the following statement on what the analyses will tell us about the nature of movie-driven activity in infants:

“These three analyses assess key indicators of the mature visual system: functional specialization between areas, organization within areas, and consistency between individuals.” Pg. 5

Furthermore, in the discussion we revisit these motivations and elaborate on them further:

[Regarding homotopy:] “This suggests that visual areas are functionally differentiated in infancy and that this function is shared across hemispheres (Yates et al., 2023).” Pg. 19

[Regarding ICA:] “This means that the retinotopic organization of the infant brain accounts for a detectable amount of variance in visual activity, otherwise components resembling these maps would not be discoverable.” Pg. 19–20

[Regarding SRM:] “This is initial evidence that functional alignment may be useful for enhancing signal quality, like it has in adults (Lu et al., 2017; Li et al., 2022; Busch et al., 2021), or revealing changing function over development (Yates et al., 2021).” Pg. 21

Additionally, we have expanded our discussion of relevant work that uses similar methods such as the excellent research from Knapen (2021) and others:

“In adults, movies and resting-state data have been used to characterize the visual cortex in a data-driven fashion (Loiotile et al., 2019; Knapen, 2021; Lu et al., 2017).” Pg. 4

“We next explored whether movies can reveal fine-grained organization within visual areas by using independent components analysis (ICA) to propose visual maps in individual infant brains (Loiotile et al., 2019; Knapen, 2021; Kumar et al., 2020; Beckmann et al., 2005; Moeller et al., 2009).” Pg. 9

**Reviewer #2 (Public Review):**

Summary:

This manuscript shows evidence from a dataset with awake movie-watching in infants, that the infant brain contains areas with distinct functions, consistent with previous studies using resting state and awake task-based infant fMRI. However, substantial new analyses would be required to support the novel claim that movie-watching data in infants can be used to identify retinotopic areas or to capture within-area functional organization.

Strengths:

The authors have collected a unique dataset: the same individual infants both watched naturalistic animations and a specific retinotopy task. These data position the authors to test their novel claim, that movie-watching data in infants can be used to identify retinotopic areas.

Weaknesses:

To claim that movie-watching data can identify retinotopic regions, the authors should provide evidence for two claims:

- Retinotopic areas defined based only on movie-watching data, predict retinotopic responses in independent retinotopy-task-driven data.

- Defining retinotopic areas based on the infant's own movie-watching response is more accurate than alternative approaches that don't require any movie-watching data, like anatomical parcellations or shared response activation from independent groups of participants.

We thank the reviewer for their comments. Before addressing their suggestions, we wish to clarify that we do not claim that movie data can be used to identify retinotopic areas, but instead that movie data captures components of the within and between visual area organization as defined by retinotopic mapping. We recognize that this was not clear in our original manuscript and have clarified this point throughout, including in this section of the discussion:

“To be clear, we are not suggesting that movies work well enough to replace a retinotopy task when accurate maps are needed. For instance, even though ICA found components that were highly correlated with the spatial frequency map, we also selected some components that turned out to have lower correlations. Without knowing the ground truth from a retinotopy task, there would be no way to weed these out. Additionally, anatomical alignment (i.e., averaging the maps from other participants and anatomically aligning them to a held-out participant) resulted in maps that were highly similar to the ground truth. Indeed, we previously (Henriksson et al., 2008) found that adult-defined visual areas were moderately similar to infants. While functional alignment with adults can outperform anatomical alignment methods in similar analyses (Lu et al., 2017), here we find that functional alignment with infants is inferior to anatomical alignment. Thus, if the goal is to define visual areas in an infant that lacks task-based retinotopy, anatomical alignment of other participants’ retinotopic maps is superior to using movie-based analyses, at least as we tested it.” Pg. 21

In response to the reviewer’s suggestion, we compare the maps identified by SRM to the averaged, anatomically aligned maps from infants. We find that these maps are highly similar to the task-based ground truth and we describe this result in a new section:

“We performed an anatomical alignment analog of the functional alignment (SRM) approach. This analysis serves as a benchmark for predicting visual maps using taskbased data, rather than movie data, from other participants. For each infant participant, we aggregated all other infant or adult participants as a reference. The retinotopic maps from these reference participants were anatomically aligned to the standard surface template, and then averaged. These averages served as predictions of the maps in the test participant, akin to SRM, and were analyzed equivalently (i.e., correlating the gradients in the predicted map with the gradients in the task-based map). These correlations (Table S4) are significantly higher than for functional alignment (using infants to predict spatial frequency, anatomical alignment < functional alignment: ∆_Fisher Z_ M=0.44, CI=[0.32–0.58], p<.001; using infants to predict meridians, anatomical alignment < functional alignment: ∆_Fisher Z_ M=0.61, CI=[0.47–0.74], p<.001; using adults to predict spatial frequency, anatomical alignment < functional alignment: ∆_Fisher Z_ M=0.31, CI=[0.21–0.42], p<.001; using adults to predict meridians, anatomical alignment < functional alignment: ∆_Fisher Z_ M=0.49, CI=[0.39–0.60], p<.001). This suggests that even if SRM shows that movies can be used to produce retinotopic maps that are significantly similar to a participant, these maps are not as good as those that can be produced by anatomical alignment of the maps from other participants without any movie data.” Pg. 16–17

Note that we do not compare the anatomically aligned maps with the ICA maps statistically. This is because these analyses are not comparable: ICA is run withinparticipant whereas anatomical alignment is necessarily between-participant — either infant or adults. Nonetheless, an interested reader can refer to the Table where we report the results of anatomical alignment and see that anatomical alignment outperforms ICA in terms of the correlation between the predicted and task-based maps.

Both of these analyses are possible, using the (valuable!) data that these authors have collected, but these are not the analyses that the authors have done so far. Instead, the authors report the inverse of (1): regions identified by the retinotopy task can be used to predict responses in the movies. The authors report one part of (2), shared responses from other participants can be used to predict individual infants' responses in the movies, but they do not test whether movie data from the same individual infant can be used to make better predictions of the retinotopy task data, than the shared response maps.

So to be clear, to support the claims of this paper, I recommend that the authors use the retinotopic task responses in each individual infant as the independent "Test" data, and compare the accuracy in predicting those responses, based on:

- The same infant's movie-watching data, analysed with MELODIC, when blind experimenters select components for the SF and meridian boundaries with no access to the ground-truth retinotopy data.

- Anatomical parcellations in the same infant.

- Shared response maps from groups of other infants or adults.

- (If possible, ICA of resting state data, in the same infant, or from independent groups of infants).

Or, possibly, combinations of these techniques.

If the infant's own movie-watching data leads to improved predictions of the infant's retinotopic task-driven response, relative to these existing alternatives that don't require movie-watching data from the same infant, then the authors' main claim will be supported.

These are excellent suggestions for additional analyses to test the suitability for moviebased maps to replace task-based maps. We hope it is now clear that it was never our intention to claim that movie-based data could replace task-based methods. We want to emphasize that the discoveries made in this paper — that movies evoke fine-grained organization in infant visual cortex — do not rely on movie-based maps being better than alternative methods for producing maps, such as the newly added anatomical alignment.

The proposed analysis above solves a critical problem with the analyses presented in the current manuscript: the data used to generate maps is identical to the data used to validate those maps. For the task-evoked maps, the same data are used to draw the lines along gradients and then test for gradient organization. For the component maps, the maps are manually selected to show the clearest gradients among many noisy options, and then the same data are tested for gradient organization. This is a double-dipping error. To fix this problem, the data must be split into independent train and test subsets.

We appreciate the reviewer’s concern; however, we believe it is a result of a miscommunication in our analytic strategy. We have now provided more details on the analyses to clarify how double-dipping was avoided.

To summarize, a retinotopy task produced visual maps that were used to trace both area boundaries and gradients across the areas. These data were then fixed and unchanged, and we make no claims about the nature of these maps in this paper, other than to treat them as the ground truth to be used as a benchmark in our analyses. The movie data, which are collected independently from the same infant in the session, used the boundaries from the retinotopy task (in the case of homotopy) or were compared with the maps from the retinotopy task (in the case of ICA and SRM). In other words, the statement that “the data used to generate maps is identical to the data used to validate those maps” is incorrect because we generated the maps with a retinotopy task and validated the maps with the movie data. This means no double dipping occurred.

Perhaps a cause of the reviewer’s interpretation is that the gradients used in the analysis are not clearly described. We now provide this additional description: “Using the same manually traced lines from the retinotopy task, we measured the intensity gradients in each component from the movie-watching data. We can then use the gradients of intensity in the retinotopy task-defined maps as a benchmark for comparison with the ICA-derived maps.” Pg. 10

Regarding the SRM analyses, we take great pains to avoid the possibility of data contamination. To emphasize how independent the SRM analysis is, the prediction of the retinotopic map from the test participant does not use their retinotopy data at all; in fact, the predicted maps could be made before that participant’s retinotopy data were ever collected. To make this prediction for a test participant, we need to learn the inversion of the SRM, but this only uses the movie data of the test participant. Hence, there is no double-dipping in the SRM analyses. We have elaborated on this point in the revision, and we remade the figure and its caption to clarify this point:

We also have updated the description of these results to emphasize how double-dipping was avoided:

“We then mapped the held-out participant's movie data into the learned shared space without changing the shared space (Figure 5c). In other words, the shared response model was learned and frozen before the held-out participant’s data was considered.

This approach has been used and validated in prior SRM studies (Yates et al., 2021).” Pg. 14

The reviewer suggests that manually choosing components from ICA is double-dipping. Although the reviewer is correct that the manual selection of components in ICA means that the components chosen ought to be good candidates, we are testing whether those choices were good by evaluating those components against the task-based maps that were not used for the ICA. Our statistical analyses evaluate whether the components chosen were better than the components that would have been chosen by random chance. Critically: all decisions about selecting the components happen before the components are compared to the retinotopic maps. Hence there is no double-dipping in the selection of components, as the choice of candidate ICA maps is not informed by the ground-truth retinotopic maps. We now clarify what the goal of this process is in the results:

“Success in this process requires that (1) retinotopic organization accounts for sufficient variance in visual activity to be identified by ICA and (2) experimenters can accurately identify these components.” Pg. 10

The reviewer also alludes to a concern that the researcher selecting the maps was not blind to the ground-truth retinotopic maps from participants and this could have influenced the results. In such a scenario, the researcher could have selected components that have the gradients of activity in the places that the infant has as ground truth. The researcher who made the selection of components (CTE) is one of the researchers who originally traced the areas in the participants approximately a year prior to the identification of ICs. The researcher selecting the components didn’t use the ground-truth retinotopic maps as reference, nor did they pay attention to the participant IDs when sorting the IC components. Indeed, they weren’t trying to find participant specific maps per se, but rather aimed to find good candidate retinotopic maps in general. In the case of the newly added adult analyses, the ICs were selected before the retinotopic mapping was reviewed or traced; hence, no knowledge about the participant-specific ground truth could have influenced the selection of ICs. Even with this process from adults, we find results of comparable strength as we found in infants, as shown below. Nonetheless, there is a possibility that this researcher’s previous experience of tracing the infant maps could have influenced their choice of components at the participant-specific level. If so, it was a small effect since the components the researcher selected were far from the best possible options (i.e., rankings of the selected components averaged in the 64th percentile for spatial frequency maps and the 68th percentile for meridian maps). We believe all reasonable steps were taken to mitigate bias in the selection of ICs.

**Reviewer #3 (Public Review):**

The manuscript reports data collected in awake toddlers recording BOLD while watching videos. The authors analyse the BOLD time series using two different statistical approaches, both very complex but do not require any a priori determination of the movie features or contents to be associated with regressors. The two main messages are that (1) toddlers have occipital visual areas very similar to adults, given that an SRM model derived from adult BOLD is consistent with the infant brains as well; (2) the retinotopic organization and the spatial frequency selectivity of the occipital maps derived by applying correlation analysis are consistent with the maps obtained by standard and conventional mapping.

Clearly, the data are important, and the author has achieved important and original results. However, the manuscript is totally unclear and very difficult to follow; the figures are not informative; the reader needs to trust the authors because no data to verify the output of the statistical analysis are presented (localization maps with proper statistics) nor so any validation of the statistical analysis provided. Indeed what I think that manuscript means, or better what I understood, may be very far from what the authors want to present, given how obscure the methods and the result presentation are.

In the present form, this reviewer considers that the manuscript needs to be totally rewritten, the results presented each technique with appropriate validation or comparison that the reader can evaluate.

We are grateful to the reviewer for the chance to improve the paper. We have broken their review into three parts: clarification of the methods, validation of the analyses, and enhancing the visualization.

Clarification of the methods

We acknowledge that the methods we employed are complex and uncommon in many fields of neuroimaging. That said, numerous papers have conducted these analyses on adults (Beckman et al., 2005; Butt et al., 2015; Guntupalli et al., 2016; Haak & Beckman, 2018; Knapen, 2021; Lu et al., 2017) and non-human primates (Arcaro & Livingstone, 2017; Moeller et al., 2009). We have redoubled our efforts in the revision to make the methods as clear as possible, expanding on the original text and providing intuitions where possible. These changes have been added throughout and are too vast in number to repeat here, especially without context, but we hope that readers will have an easier time following the analyses now.

Additionally, we updated Figures 3 and 5 in which the main ICA and SRM analyses are described. For instance, in Figure 3’s caption we now add details about how the gradient analyses were performed on the components:

“We used the same lines that were manually traced on the task-evoked map to assess the change in the component’s response. We found a monotonic trend within area from medial to lateral, just like we see in the ground truth.” Pg. 11

Regarding Figure 5, we reconsidered the best way to explain the SRM analyses and decided it would be helpful to partition the diagram into steps, reflecting the analytic process. These updates have been added to Figure 5, and the caption has been updated accordingly.

We hope that these changes have improved the clarity of the methods. For readers interested in learning more, we encourage them to either read the methods-focused papers that debut the analyses (e.g., Chen et al., 2015), read the papers applying the methods (e.g., Guntupalli et al., 2016), or read the annotated code we publicly release which implements these pipelines and can be used to replicate the findings.

Validation of the analyses

One of the requests the reviewer makes is to validate our analyses. Our initial approach was to lean on papers that have used these methods in adults or primates (e.g., Arcaro, & Livingstone, 2017; Beckman et al., 2005; Butt et al., 2015; Guntupalli et al., 2016; Haak & Beckman, 2018; Knapen, 2021; Moeller et al., 2009) where the underlying organization and neurophysiology is established. However, we have made changes to these methods that differ from their original usage (e.g., we used SRM rather than hyperalignment, we use meridian mapping rather than traveling wave retinotopy, we use movie-watching data rather than rest). Hence, the specifics of our design and pipeline warrant validation.

To add further validation, we have rerun the main analyses on an adult sample. We collected 8 adult participants who completed the same retinotopy task and a large subset of the movies that infants saw. These participants were run under maximally similar conditions to infants (i.e., scanned using the same parameters and without the top of the head-coil) and were preprocessed using the same pipeline. Given that the relationship between adult visual maps and movie-driven (or resting-state) analyses has been shown in many studies (Beckman et al., 2005; Butt et al., 2015; Guntupalli et al., 2016; Haak & Beckman, 2018; Knapen, 2021; Lu et al., 2017), these adult data serve as a validation of our analysis pipeline. These adult participants were included in the original manuscript; however, they were previously only used to support the SRM analyses (i.e., can adults be used to predict infant visual maps). The adult results are described before any results with infants, as a way to engender confidence. Moreover, we have provided new supplementary figures of the adult results that we hope will be integrated with the article when viewing it online, such that it will be easy to compare infant and adult results, as per the reviewer’s request.

As per the figures and captions below, the analyses were all successful with the adult participants: (1) Homotopic correlations are higher than correlations between comparable areas in other streams or areas that are more distant within stream. (2) A multidimensional scaling depiction of the data shows that areas in the dorsal and ventral stream are dissimilar. (3) Using independent components analysis on the movie data, we identified components that are highly correlated with the retinotopy task-based spatial frequency and meridian maps. (4) Using shared response modeling on the movie data, we predicted maps that are highly correlated with the retinotopy task-based spatial frequency and meridian maps.

These supplementary analyses are underpowered for between-group comparisons, so we do not statistically compare the results between infants and adults. Nonetheless, the pattern of adult results is comparable overall to the infant results.

We believe these adult results provide a useful validation that the infant analyses we performed can recover fine-grained organization.

Enhancing the visualization

The reviewer raises an additional concern about the lack of visualization of the results. We recognize that the plots of the summary statistics do not provide information about the intermediate analyses. Indeed, we think the summary statistics can understate the degree of similarity between the components or predicted visual maps and the ground truth. Hence, we have added 6 new supplementary figures showing the intensity gradients for the following analyses: 1. spatial frequency prediction using ICA, 2. meridian prediction using ICA, 3. spatial frequency prediction using infant SRM, 4. meridian prediction using infant SRM, 5. spatial frequency prediction using adult SRM, and 6. meridian prediction using adult SRM.

We hope that these visualizations are helpful. It is possible that the reviewer wishes us to also visually present the raw maps from the ICA and SRM, akin to what we show in Figure 3A and 3B. We believe this is out of scope of this paper: of the 1140 components that were identified by ICA, we selected 36 for spatial frequency and 17 for meridian maps. We also created 20 predicted maps for spatial frequency and 20 predicted meridian maps using SRM. This would result in the depiction of 93 subfigures, requiring at least 15 new full-page supplementary figures to display with adequate resolution. Instead, we encourage the reader to access this content themselves: we have made the code to recreate the analyses publicly available, as well as both the raw and preprocessed data for these analyses, including the data for each of these selected maps.

**Recommendations for the authors:**

**Reviewer #1 (Recommendations For The Authors):**

(1) As mentioned in the public review, the authors should consider incorporating relevant adult fMRI research into the Introduction and explain the importance of testing this question in infants.

Our public response describes the several citations to relevant adult research we have added, and have provided further motivation for the project.

(2) The authors should conduct additional analyses to support their conclusion that movie data alone can generate accurate retinotopic maps (i.e., by comparing this approach to other available alternatives).

We have clarified in our public response that we did not wish to conclude that movie data alone can generate accurate retinotopic maps, and have made substantial edits to the text to emphasize this. Thus, because this claim is already not supported by our analyses, we do not think it is necessary to test it further.

(3) The authors should re-do the homotopy analyses using movie-defined ROIs (i.e., by splitting the movie-viewing data into independent folds for functional ROI definition and analyses).

As stated above, defining ROIs based on the movie content is not the intended goal of this project. Even if that were the general goal, we do not believe that it would be appropriate to run this specific analysis with the data we collected. Firstly, halving the data for ROI definition (e.g., using half the movie data to identify and trace areas, and then use those areas in the homotopy analysis to run on the other half of data) would qualitatively change the power of the analyses described here. Secondly, we would be unable to define areas beyond hV4/V3AB with confidence, since our retinotopic mapping only affords specification of early visual cortex. Thus we could not conduct the MDS analyses shown in Figure 2.

(4) If the authors agree that a primary contribution of this study and paper is to showcase what is possible to do with a limited amount of movie-viewing data, then they should make it clearer, sooner, how much usable movie data they have from infants. They could also consider conducting additional analyses to determine the minimum amount of fMRI data necessary to reveal the same detailed characteristics of functional responses in the visual cortex.

We agree it would be good to highlight the amount of movie data used. When the infant data is first introduced in the results section, we now state the durations:

“All available movies from each session were included (Table S2), with an average duration of 540.7s (range: 186-1116s).” Pg. 5

Additionally, we have added a homotopy analysis that describes the contribution of data quantity to the results observed. We compare the amount of data collected with the magnitude of same vs. different stream effect (Figure 1B) and within stream distance effect (Figure 1C). We find no effect of movie duration in the sample we tested, as reported below:

“We found no evidence that the variability in movie duration per participant correlated with this difference [of same stream vs. different stream] (r=0.08, p=.700).” Pg. 6-7

“There was no correlation between movie duration and the effect (Same > Adjacent: r=-0.01, p=.965, Adjacent > Distal: r=-0.09, p=.740).” Pg. 7

(5) If any of the methodological approaches are novel, the authors should make this clear. In particular, has the approach of visually inspecting and categorizing components generated from ICA and movie data been done before, in adults/other contexts?

The methods we employed are similar to others, as described in the public review.

However, changes were necessary to apply them to infant samples. For instance, Guntupalli et al. (2016) used hyperalignment to predict the visual maps of adult participants, whereas we use SRM. SRM and hyperalignment have the same goal — find a maximally aligned representation between participants based on brain function — but their implementation is different. The application of functional alignment to infants is novel, as is their use in movie data that is relatively short by comparison to standard adult data. Indeed, this is the most thorough demonstration that SRM — or any functional alignment procedure — can be usefully applied to infant data, awake or sleeping. We have clarified this point in the discussion.

“This is initial evidence that functional alignment may be useful for enhancing signal quality, like it has in adults (Lu et al., 2017; Li et al., 2022; Busch et al., 2021), or revealing changing function over development (Yates et al., 2021), which may prove especially useful for infant fMRI (Ellis and Turk-Browne, 2018).” Pg. 21

(6) The authors found that meridian maps were less identifiable from ICA and movie data and suggest that this may be because these maps are more susceptible to noise or gaze variability. If this is the case, you might predict that these maps are more identifiable in adult data. The authors could consider running additional analyses with their adult participants to better understand this result.

As described in the manuscript, we hypothesize that meridian maps are more difficult to identify than spatial frequency maps because meridian maps are a less smooth, more fine-grained map than spatial frequency. Indeed, it has previously been reported (Moeller et al., 2009) that similar procedures can result in meridian maps that are constituted by multiple independent components (e.g., a component sensitive to horizontal orientations, and a separate component sensitive to vertical components). Nonetheless, we have now conducted the ICA procedure on adult participants and again find it is easier to identify spatial frequency components compared to meridian maps, as reported in the public review.

Minor corrections:

(1) Typo: Figure 3 title: "Example retintopic task vs. ICA-based spatial frequency maps.".

Fixed

(2) Given the age range of the participants, consider using "infants and toddlers"? (Not to diminish the results at all; on the contrary, I think it is perhaps even more impressive to obtain awake fMRI data from ~1-2-year-olds). Example: Figure 3 legend: "(A) Spatial frequency map of a 17.1-monthold infant.".

We agree with the reviewer that there is disagreement about the age range at which a child starts being considered a toddler. We have changed the terms in places where we refer to a toddler in particular (e.g., the figure caption the reviewer highlights) and added the phrase “infants and toddlers” in places where appropriate. Nonetheless, we have kept “infants” in some places, particularly those where we are comparing the sample to adults. Adding “and toddlers” could imply three samples being compared which would confuse the reader.

(3) Figure 6 legend: The following text should be omitted as there is no bar plot in this figure: "The bar plot is the average across participants. The error bar is the standard error across participants.".

Fixed

(4) Table S1 legend: Missing first single quote: Runs'.

Fixed

**Reviewer #2 (Recommendations For The Authors):**

I request that this paper cite more of the existing literature on the fMRI of human infants and toddlers using task-driven and resting-state data. For example, early studies by (first authors) Biagi, Dehaene-Lambertz, Cusack, and Fransson, and more recent studies by Chen, Cabral, Truzzi, Deen, and Kosakowski.

We have added several new citations of recent task-based and resting state studies to the second sentence of the main text:

“Despite the recent growth in infant fMRI (Biagi et al., 2015; Biagi et al., 202 3; Cabral et al, 2022; Deen et al., 2017; Kosakowski et al., 2021; Truzzi and Cusack, 2023), one of the most important obstacles facing this research is that infants are unable to maintain focus for long periods of time and struggle to complete traditional cognitive tasks (Ellis et al., 2020).”

**Reviewer #3 (Recommendations For The Authors):**

In the following, I report some of my main perplexities, but many more may arise when the material is presented more clearly.

The age of the children varies from 5 months to about 2 years. While the developmental literature suggests that between 1 and 2 years children have a visual system nearly adult-like, below that age some areas may be very immature. I would split the sample and perhaps attempt to validate the adult SRM model with the youngest children (and those can be called infants).

We recognize the substantial age variability in our sample, which is why we report participant-specific data in our figures. While splitting up the data into age bins might reveal age effects, we do not think we can perform adequately powered null hypothesis testing of the age trend. In order to investigate the contribution of age, larger samples will be needed. That said, we can see from the data that we have reported that any effect of age is likely small. To elaborate: Figures 4 and 6 report the participant-specific data points and order the participants by age. There are no clear linear trends in these plots, thus there are no strong age effects.

More broadly, we do not think there is a principled way to divide the participants by age. The reviewer suggests that the visual system is immature before the first year of life and mature afterward; however, such claims are the exact motivation for the type of work we are doing here, and the verdict is still out. Indeed, the conclusion of our earlier work reporting retinotopy in infants (Ellis et al., 2021) suggests that the organization of the early visual cortex in infants as young as 5 months — the youngest infant in our sample — is surprisingly adult-like.

The title cannot refer to infants given the age span.

There is disagreement in the field about the age at which it is appropriate to refer to children as infants. In this paper, and in our prior work, we followed the practice of the most attended infant cognition conference and society, the International Congress of Infant Studies (ICIS), which considers infants as those aged between 0-3 years old, for the purposes of their conference. Indeed, we have never received this concern across dozens of prior reviews for previous papers covering a similar age range. That said, we understand the spirit of the reviewer’s comment and now refer to the sample as “infants and toddlers” and to older individuals in our sample as “toddlers” wherever it is appropriate (the younger individuals would fairly be considered “infants” under any definition).

Figure 1 is clear and an interesting approach. Please also show the average correlation maps on the cortical surface.

While we would like to create a figure as requested, we are unsure how to depict an area-by-area correlation map on the cortical surface. One option would be to generate a seed-based map in which we take an area and depict the correlation of that seed (e.g., vV1) with all other voxels. This approach would result in 8 maps for just the task-defined areas, and 17 maps for anatomically-defined areas. Hence, we believe this is out of scope of this paper, but an interested reader could easily generate these maps from the data we have released.

Figure 2 results are not easily interpretable. Ventral and dorsal V1-V3 areas represent upper or lower VF respectively. Higher dorsal and ventral areas represent both upper and lower VF, so we should predict an equal distance between the two streams. Again, how can we verify that it is not a result of some artifacts?

In adults, visual areas differ in their functional response properties along multiple dimensions, including spatial coding. The dorsal/ventral stream hypothesis is derived from the idea that areas in each stream support different functions, independent of spatial coding. The MDS analysis did not attempt to isolate the specific contribution of spatial representations of each area but instead tested the similarity of function that is evoked in naturalistic viewing. Other covariance-based analyses specifically isolate the contribution of spatial representations (Haak et al., 2013); however, they use a much more constrained analysis than what was implemented here. The fact that we find broad differentiation of dorsal and ventral visual areas in infants is consistent with adults (Haak & Beckman, 2018) and neonate non-human primates (Arcaro & Livingstone, 2017).

Nonetheless, we recognize that we did not mention the differences in visual field properties across areas and what that means. If visual field properties alone drove the functional response then we would expect to see a clustering of areas based on the visual field they represent (e.g., hV4 and V3AB should have similar representations). Since we did not see that, and instead saw organization by visual stream, the result is interesting and thus warrants reporting. We now mention this difference in visual fields in the manuscript to highlight the surprising nature of the result.

“This separation between streams is striking when considering that it happens *despite* differences in visual field representations across areas: while dorsal V1 and ventral V1 represent the lower and upper visual field, respectively, V3A/B and hV4 both have full visual field maps. These visual field representations can be detected in adults (Haak et al., 2013); however, they are often not the primary driver of function (Haak and Beckmann, 2018). We see that in infants too: hV4 and V3A/B represent the same visual space yet have distinct functional profiles.” Pg. 8

The reviewer raises a concern that the MDS result may be spurious and caused by noise. Below, we present three reasons why we believe these results are not accounted for by artifacts but instead reflect real functional differentiation in the visual cortex.

(1) Figure 2 is a visualization of the similarity matrix presented in Figure S1. In Figure S1, we report the significance testing we performed to confirm that the patterns differentiating dorsal and ventral streams — as well as adjacent areas from distal areas — are statistically reliable across participants. If an artifact accounted for the result then it would have to be a kind of systematic noise that is consistent across participants.

(2) One of the main sources of noise (both systematic and non-systematic) with infant fMRI is motion. Homotopy is a within-participant analysis that could be biased by motion. To assess whether motion accounts for the results, we took a conservative approach of regressing out the framewise motion (i.e., how much movement there is between fMRI volumes) from the comparisons of the functional activity in regions. Although the correlations numerically decreased with this procedure, they were qualitatively similar to the analysis that does not regress out motion:

“Additionally, if we control for motion in the correlation between areas – in case motion transients drive consistent activity across areas – then the effects described here are negligibly different (Figure S5).” Pg. 7

(3) We recognize that despite these analyses, it would be helpful to see what this pattern looks like in adults where we know more about the visual field properties and the function of dorsal and ventral streams. This has been done previously (e.g., Haak & Beckman, 2018), but we have now run those analyses on adults in our sample, as described in the public review. As with infants, there are reliable differences in the homotopy between streams (Figure S1). The MDS results show that the adult data was more complex than the infant data, since it was best described by 3 dimensions rather than 2. Nonetheless, there is a rotation of the MDS such that the structure of the ventral and dorsal streams is also dissociable.

Figure 3 also raises several alternative interpretations. The spatial frequency component in B has strong activity ONLY at the extreme border of the VF and this is probably the origin of the strong correlation. I understand that it is only one subject, but this brings the need to show all subjects and to report the correlation. Also, it is important to show the putative average ICA for retinotopy and spatial frequencies across subjects and for adults. All methods should be validated on adults where we have clear data for retinotopy and spatial frequency.

The reviewer notes that the component in Figure 3 shows strong negative response in the periphery. It is often the case, as reported elsewhere (Moeller et al., 2009), that ICA extracts portions of visual maps. To make a full visual map would require combining components into a composite (e.g., a component that has a high response in the periphery and another component that has a high response in the fovea). If we were to claim that this component, or others like it, could replace the need for retinotopic mapping, then we would want to produce these composite maps; however, our conclusion in this project is that the topographic information of retinotopic maps manifest in individual components of ICA. For this purpose, the analysis we perform adequately assesses this topography.

Regarding the request to show the results for all subjects, we address this in the public response and repeat it here briefly: we have added 6 new figures to show results akin to Figure 3C and D. It is impractical to show the equivalent of Figure 3A and B for all participants, yet we do release the data necessary to see to visualize these maps easily.

Finally, the reviewer suggests that we validate the analyses on adult participants. As shown in Figure S3 and reported in the public response, we now run these analyses on adult participants and observe qualitatively similar results to infants.

How much was the variation in the presumed spatial frequency map? Is it consistent with the acuity range? 5-month-old infants should have an acuity of around 10c/deg, depending on the mean luminance of the scene.

The reviewer highlights an important weakness of conducting ICA: we cannot put units on the degree of variation we see in components. We now highlight this weakness in the discussion:

“Another limitation is that ICA does not provide a scale to the variation: although we find a correlation between gradients of spatial frequency in the ground truth and the selected component, we cannot use the component alone to infer the spatial frequency selectivity of any part of cortex. In other words, we cannot infer units of spatial frequency sensitivity from the components alone.” Pg. 20

Figure 5 pipeline is totally obscure. I presumed that I understood, but as it is it is useless. All methods should be clearly described, and the intermediate results should be illustrated in figures and appropriately discussed. Using such blind analyses in infants in principle may not be appropriate and this needs to be verified. Overall all these techniques rely on correlation activities that are all biased by head movement, eye movement, and probably the dummy sucking. All those movements need to be estimated and correlated with the variability of the results. It is a strong assumption that the techniques should work in infants, given the presence of movements.

We recognize that the SRM methods are complex. Given this feedback, we remade Figure 5 with explicit steps for the process and updated the caption (as reported in the public review).

Regarding the validation of these methods, we have added SRM analyses from adults and find comparable results. This means that using these methods on adults with comparable amounts of data as what we collected from infants can predict maps that are highly similar to the real maps. Even so, it is not a given that these methods are valid in infants. We present two considerations in this regard.

First, as part of the SRM analyses reported in the manuscript, we show that control analyses are significantly worse than the real analyses (indicated by the lines on Figure 6). To clarify the control analysis: we break the mapping (i.e., flip the order of the data so that it is backwards) between the test participant and the training participants used to create the SRM. The fact that this control analysis is significantly worse indicates that SRM is learning meaningful representations that matter for retinotopy.

Second, we believe that this paper is a validation of SRM for infants. Infant fMRI is a nascent field and SRM has the potential to increase the signal quality in this population. We hope that readers will see these analyses as a proof of concept that SRM can be used in their work with infants. We have stated this contribution in the paper now.

“Additionally, we wish to test whether methods for functional alignment can be used with infants. Functional alignment finds a mapping between participants using functional activity – rather than anatomy – and in adults can improve signal-to-noise, enhance across participant prediction, and enable unique analyses (Lu et al., 2017; Li et al., 2022; Busch et al., 2021; Chen et al., 2015).” Pg. 4

“This is initial evidence that functional alignment may be useful for enhancing signal quality, like it has in adults (Lu et al., 2017; Li et al., 2022; Busch et al., 2021), or revealing changing function over development (Yates et al., 2021).” Pg. 21

Regarding the reviewer’s concern that motion may bias the results, we wish to emphasize the nature of the analyses being conducted here: we are using data from a group of participants to predict the neural responses in a held-out participant. For motion to explain consistency between participants, the motion would need to be timelocked across participants. Even if motion was time-locked during movie watching, motion will impair the formation of an adequate model that can contain retinotopic information. Thus, motion should only hurt the ability for a shared response to be found that can be used for predicting retinotopic maps. Hence, the results we observed are despite motion and other sources of noise.

What is M??? is it simply the mean value??? If not, how it is estimated?

M is an abbreviation for mean. We have now expanded the abbreviation the first time we use it.

Figure 6 should be integrated with map activity where the individual area correlation should be illustrated. Probably fitting SMR adult works well for early cortical areas, but not for more ventral and associative, and the correlation should be evaluated for the different masks.

With the addition of plots showing the gradients for each participant and each movie (Figures S10–S13) we hope we have addressed this concern. We additionally want to clarify that the regions we tested in the analysis in Figure 6 are only the early visual areas V1, V2, V3, V3A/B, and hV4. The adult validation analyses show that SRM works well for predicting the visual maps in these areas. Nonetheless, it is an interesting question for future research with more extensive retinotopic mapping in infants to see if SRM can predict maps beyond extrastriate cortex.

Occipital masks have never been described or shown.

The occipital mask is from the MNI probabilistic structural atlas (Mazziotta et al., 2001), as reported in the original version and is shared with the public data release. We have added the additional detail that the probabilistic atlas is thresholded at 0% in order to be liberally inclusive.

“We used the occipital mask from the MNI structural atlas (Mazziotta et al., 2001) in standard space – defined liberally to include any voxel with an above zero probability of being labelled as the occipital lobe – and used the inverted transform to put it into native functional space.” Pg. 27–28

Methods lack the main explanation of the procedures and software description.

We hope that the additions we have made to address this reviewer’s concerns have provided better explanations for our procedures. Additionally, as part of the data and code release, we thoroughly explain all of the software needed to recreate the results we have observed here.
